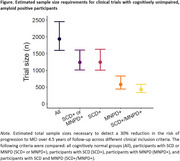# Subjective Cognitive Decline and Minor Neuropsychological Deficits: Importance for Risk Stratification and Trial Recruitment in Early Alzheimer´s Disease

**DOI:** 10.1002/alz70857_102815

**Published:** 2025-12-25

**Authors:** Melina Stark, Elizabeth Kuhn, Michael Wagner, Henning Boecker, Frederic Brosseron, Katharina Buerger, Marcel Daamen, Christoph Laske, Robert Perneczky, Oliver Peters, Josef Priller, Matthias Schmid, Anja Schneider, Annika Spottke, Stefan J. Teipel, Jens Wiltfang, Emrah Düzel, Frank Jessen, Luca Kleineidam

**Affiliations:** ^1^ Department for Old Age Psychiatry and Cognitive Disorders, University Hospital Bonn, Bonn, Germany; ^2^ German Center for Neurodegenerative Diseases (DZNE), Bonn, Germany; ^3^ Clininical Functional Imaging Group, Department of Diagnostic and Interventional Radiology, University Hospital Bonn, Bonn, Germany; ^4^ Institute for Stroke and Dementia Research (ISD), University Hospital, LMU, Munich, Germany; ^5^ German Center for Neurodegenerative Diseases (DZNE), Munich, Germany; ^6^ German Center for Neurodegenerative Diseases (DZNE), Tübingen, Germany; ^7^ Section for Dementia Research, Hertie Institute for Clinical Brain Research and Department of Psychiatry and Psychotherapy, University of Tübingen, Tübingen, Germany; ^8^ Ageing Epidemiology (AGE) Research Unit, School of Public Health, Imperial College London, London, United Kingdom; ^9^ Munich Cluster for Systems Neurology (SyNergy), Munich, Germany; ^10^ Department of Psychiatry and Psychotherapy, University Hospital, LMU Munich, Munich, Germany; ^11^ Department of Psychiatry and Neurosciences, Charité Universitätsmedizin Berlin, Berlin, Germany; ^12^ Charité Universitätsmedizin Berli, Experimental and Clinical Research Center (ECRC), Berlin, Germany; ^13^ German Center for Neurodegenerative Diseases (DZNE), Berlin, Germany; ^14^ Department of Psychiatry and Psychotherapy, School of Medicine and Health, Technical University of Munich, and German Center for Mental Health (DZPG), Munich, Germany; ^15^ University of Edinburgh and UK DRI, Edinburgh, United Kingdom; ^16^ Department of Psychiatry and Psychotherapy, Charité, Berlin, Germany; ^17^ Institute for Medical Biometry, Informatics and Epidemiology, University Hospital Bonn, Bonn, Germany; ^18^ Department of Old Age Psychiatry and Cognitive Disorders, University Hospital Bonn and University of Bonn, Bonn, Germany; ^19^ Department of Neurology, University of Bonn, Bonn, Germany; ^20^ German Center for Neurodegenerative Diseases (DZNE), Venusberg‐Campus 1, 53127, Bonn, Germany; ^21^ German Center for Neurodegenerative Diseases (DZNE), Rostock, Germany; ^22^ Department of Psychosomatic Medicine, Rostock University Medical Center, Rostock, Germany; ^23^ German Center for Neurodegenerative Diseases (DZNE), Göttingen, Germany; ^24^ Department of Psychiatry and Psychotherapy, University Medical Center Goettingen, University of Goettingen, Goettingen, Germany; ^25^ Neurosciences and Signaling Group, Institute of Biomedicine (iBiMED), Department of Medical Sciences, University of Aveiro, Aveiro, Portugal; ^26^ German Center for Neurodegenerative Diseases (DZNE), Magdeburg, Germany; ^27^ Institute of Cognitive Neurology and Dementia Research (IKND), Otto‐von‐Guericke University, Magdeburg, Sachsen Anhalt, Germany; ^28^ Excellence Cluster on Cellular Stress Responses in Aging‐Associated Diseases (CECAD), University of Cologne, Cologne, Germany; ^29^ Department of Psychiatry, Medical Faculty, University of Cologne, Cologne, Germany

## Abstract

**Background:**

The recruitment of participants with a high risk of decline is crucial for the success of clinical trials in the earliest phases of Alzheimer´s Disease (AD), where treatment benefits could be the largest. Subjective cognitive decline (SCD) and minor neuropsychological deficits (MNPD) are associated with an increased risk of cognitive decline, making them promising predictors for this risk stratification. However, the prognostic value of their interplay is understudied.

**Method:**

We pooled and analyzed data from cognitively unimpaired participants from the Alzheimer´s Disease Neuroimaging Initiative (*N* = 599), DZNE Longitudinal Cognitive Impairment and Dementia study (*N* = 618), and National Alzheimer's Coordinating Center (*N* = 11,975). SCD was measured using questionnaires or anamnestic data. MNPD was defined as a median neuropsychological test performance of z≤‐0.5. We assessed the association of MNPD and SCD with the conversion to mild cognitive impairment (MCI) and dementia (cox regression) and baseline amyloid and tau positivity – measured by PET/CSF (logistic regression). We adjusted these models for the study cohorts and demographic covariates. Using power analyses, we calculated the sample sizes necessary to detect a 30% reduction in the risk of progressing to MCI over 4.5 years in amyloid positive participants.

**Result:**

In the overall sample (*N* = 13,192), the SCD‐/MNPD+ (+:present, ‐:absent; HR=3.13[2.68‐3.66]), SCD+/MNPD‐ (HR=1.97[1.76‐2.20]), and SCD+/MNPD+ (HR=6.23[5.23‐7.42]) groups had an increased risk of MCI compared to the SCD‐/MNPD‐ group. These groups also had an increased risk of dementia. In amyloid positive participants (*n* = 497), this pattern persisted for the progression to MCI, while only the SCD+/MNPD+ group had an increased risk for dementia. In participants with biomarker data (*n* = 2,616), the SCD+/MNPD‐ (OR=1.47[1.20‐1.81]) and SCD+/MNPD+ (OR=1.64[1.04‐2.59]) groups had an increased risk of amyloid positivity. The risk of tau positivity was increased in the SCD+/MNPD+ group (OR=2.10[1.13‐3.90]). In the power analyses, the required clinical trial size was reduced by approximately one third after excluding SCD‐/MNPD‐ individuals and approximately two thirds by focusing only on SCD+/MNPD+ individuals (Figure).

**Conclusion:**

SCD and MNPD have a complementary prognostic value. SCD+/MNPD+ individuals are at particularly high risk of pathology and decline. These clinical symptoms should be taken into account in the recruitment for clinical trials in preclinical AD.